# Supplementary material for: Fast-Response Photodetector Based on Hybrid Bi2Te3/PbS Colloidal Quantum Dots
Source: Nanomaterials (Basel). 2022 Sep 16;12(18):3212. doi: 10.3390/nano12183212 (PMC9506398; doi:10.3390/nano12183212)
Supplement: Supplementary file 1 [file nanomaterials-12-03212-s001.zip › nanomaterials-1865798-supplementary.pdf]

nanomaterials

Article

Fast-Response Photodetector Based on Hybrid Bi_2Te_3/PbS Colloidal Quantum Dots

Lijing Yu [1,2,3], Pin Tian [2,3], Libin Tang [1,2,3,*], Qun Hao [1,*], Kar Seng Teng [4,*], Hefu Zhong [5], Biao Yue [2], Haipeng Wang [2] and Shunying Yan [2]

[1] School of Optics and Photonics, Beijing Institute of Technology, Beijing 100081, China; yulijing29@163.com
[2] Kunming Institute of Physics, Kunming 650223, China; ynutxp@163.com (P.T.); yuebiaonwpu@yeah.net (B.Y.); Whpeng@163.com (H.W.); ysy2656@163.com (S.Y.)
[3] Yunnan Key Laboratory of Advanced Photoelectronic Materials & Devices, Kunming 650223, China
[4] Department of Electronic and Electrical Engineering, Swansea University, Bay Campus, Fabian Way, Swansea SA1 8EN, UK
[5] School of Materials and Energy, Yunnan University, Kunming 650500, China; ynuzhf@163.com
* Correspondence: scitang@163.com (L.T.); qhao@bit.edu.cn (Q.H.); k.s.teng@swansea.ac.uk (K.S.T.)

UV-Vis absorption spectrum of PbS CQDs

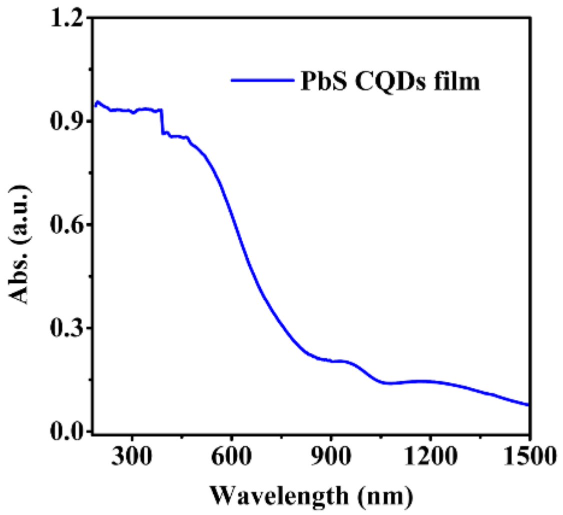

Figure S1. UV-Vis absorption spectrum of PbS CQDs.

As shown in Figure S1, PbS CQDs exhibited strong absorption from ultraviolet to near infrared hence they are highly suitable for the fabrication of multi-band photodetectors.

I-V characteristics of the device under dark and light illumination at 365, 500 and 850 nm

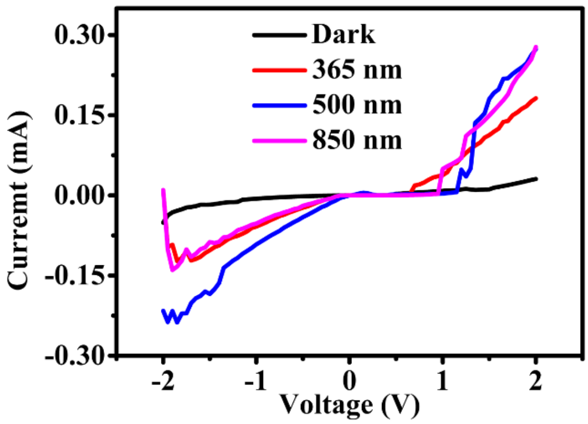

Figure S2. *I-V* plots under dark and light illumination at 365, 500 and 850 nm.

The device demonstrated obvious response under 365, 500 and 850 nm incident light as shown in Figure S2.
